# Emerging Collar Rot of Young Table Grapevines Associated with *Pleiocarpon* sp. in Coastal Peru

**DOI:** 10.3390/ijms27167448

**Published:** 2026-08-20

**Authors:** Luis A. Álvarez, Gabriela Salcedo-Astorima, Phillip Ormeño-Vásquez, Naysha Rojas-Villa, José Soto-Heredia

**Affiliations:** 1 Facultad de Ciencias Agrarias, Universidad Nacional de Cañete, Jr. San Agustín 124, San Vicente de Cañete, Cañete, Lima 15701, Peru; 2 Grupo Agrobiotecnológico de la Costa Peruana, Universidad Nacional de Cañete, Jr. San Agustín 124, San Vicente de Cañete, Cañete, Lima 15701, Peru; 3 Facultad de Agronomía y Sistemas Naturales, Pontificia Universidad Católica de Chile, Santiago 7820436, Chile; 4 Facultad de Ciencias Biológicas, Pontificia Universidad Católica de Chile, Santiago 8331150, Chile; 5 Departamento Académico de Fitopatología, Facultad de Agronomía, Universidad Nacional Agraria La Molina, La Molina, Lima 15026, Peru

**Keywords:** *Cylindrocarpon*-like morph, black foot disease, Salt Creek rootstock, *Vitis vinifera*

## Abstract

Severe decline and death of young table grapevines (1 to 2 years old) have been observed recurrently in commercial vineyards in Peru since 2022. Affected plants developed rapid shoot wilting associated with extensive necrotic lesions at the rootstock collar below the graft union, leading to plant death within days of symptom onset. A *Cylindrocarpon*-like fungus was consistently isolated from symptomatic collar tissues. Morphological characterization, cardinal temperature assays, and phylogenetic analyses based on the internal transcribed spacer (ITS) region and histone H3 (*his3*) gene identified the pathogen as a member of the genus *Pleiocarpon*. Bayesian inference of concatenated sequences resolved the Peruvian isolates as a lineage sister to *P. strelitziae* (posterior probability = 1.00). Greenhouse pathogenicity tests with two representative isolates on cv. Red Globe grafted onto Salt Creek rootstock reproduced collar lesions and shoot wilting, fulfilling Koch’s postulates. Optimal mycelial growth was estimated at 28.7–29.3 °C, with maximum colony growth recorded at 30 °C, consistent with the warm conditions that prevail during vineyard establishment in coastal Peru. Designated here as collar rot, the syndrome represents an atypically severe form of black foot disease, characterized by extensive belowground collar necrosis and rapid vine collapse soon after planting. Its recurrence across regions and seasons, together with the near-exclusive deployment of a single rootstock (Salt Creek), indicates that *Pleiocarpon*-associated collar rot is an emerging threat to young table grape plantings in Peru, underscoring the need for early diagnosis, pathogen-free nursery material, and preventive management during vineyard establishment.

## 1. Introduction

Grapevine (*Vitis vinifera* L.) is extensively cultivated in Peru, mainly for table grapes, wine production, and for the distillation of grape brandy known as “Pisco” [1]. Over recent decades, Peru has consolidated its position as the leading global exporter of table grapes for the third consecutive year, with export volumes achieving 870,000 t during the 2025–2026 campaign [2]. National production is mainly concentrated in the coastal regions of Ica and Piura, which account for 49% and 36% of the national volume, respectively [3]. This export campaign generally extends from October to April and includes more than 56 varieties of table grapes.

In late summer 2022, symptoms of decline and death of young grapevine plants (1–2 years after planting) of unknown etiology were observed in commercial vineyards in the Piura region. Affected plants exhibited wilting and eventually died, resulting in severe plant losses in the fields. Necrotic lesions were regularly observed belowground at the rootstock collar, particularly in the buried portion below the graft union. Because such collar lesions are uncommon in grapevines, this condition was provisionally designated as collar rot of grapevines.

In subsequent years, similar symptoms were observed during late spring and, more frequently, throughout the summer in Piura and other commercial grape-growing regions of Peru. However, the impact of this syndrome on young grapevine plants has not yet been thoroughly investigated.

Soil-borne pathogens, such as *Fusarium* spp., are associated with root and crown rot, vascular wilt, basal rot, and wood cankers in declining grapevines in both young and mature vineyards worldwide [4,5,6].

Spies et al. [7] reported the pathogenicity of oomycetes, including *Pythium* (*P. vexans*, *P. ultimum*, and *P. irregulare*) and *Phytophthora* (*Ph. cinnamomi* and *Ph. niederhauserii*), which are associated with crown and root rot of grapevines in South Africa. In that study, isolates of *Ph. cinnamomi* were the most aggressive, followed by those of *Ph. niederhauserii* and *P. vexans*.

In Peru, table grape production is concentrated along the hyper-arid Pacific coast, where desert conditions and extremely low annual rainfall prevail [8]. Abiotic stress factors, particularly drought and high temperatures, predispose grapevines to several economically important diseases that affect both their productivity and longevity. Among these, grapevine trunk diseases are a constant threat [9,10]. In Peru, this complex of fungal diseases, including pathogens associated with *Botryosphaeria* dieback, Petri disease, and black foot disease, has been reported in several grape-producing areas along the coastal region [11,12].

The occurrence of this disease across different regions and over multiple years, together with the observed high plant mortality, underscore the importance of investigating its etiology. Disease outbreaks could be influenced by interactions between environmental conditions and host susceptibility; therefore, accurate identification of causal agents is essential for developing effective integrated disease management strategies.

Although this syndrome has been observed repeatedly in commercial vineyards, its causal agent has not been established in Peru. Moreover, while black foot disease and other *Cylindrocarpon*-like fungi have been documented in South American vineyards, an extensive belowground collar-rot presentation of this severity and rapid lethality has not, to our knowledge, been reported in young table grapevines in the region. The present study was therefore undertaken to identify the causal agent of this rapid decline through a polyphasic approach, combining morphological characterization, cardinal-temperature assays to describe the pathogen’s thermal response, and phylogenetic analyses based on the internal transcribed spacer (ITS) and histone H3 (*his3*) regions. Greenhouse pathogenicity tests were then conducted to confirm the role of the isolates in the observed decline and to fulfill Koch’s postulates. By integrating these lines of evidence, the study aims to define the etiology of this emerging collar rot and to inform early diagnosis, nursery sanitation, and preventive management during the establishment of young table grape vineyards under warm coastal conditions.

## 2. Materials and Methods

### 2.1. Field Survey and Isolation

Symptomatic grapevine plants (1- to 2-year-old) were observed from 2022 to 2025, and diseased plant samples (n = 64) were collected from 22 different grapevine commercial orchards in the Piura Region: Piura (12), Sullana (6), Morropón (2), and two (2) grapevine fields from the Ica Region.

In each affected field, disease occurrence was estimated by recording the number of symptomatic and dead plants; these field-based counts provide an indication of disease pressure rather than the output of a formal epidemiological survey. At least two plants per orchard were collected and transported to the laboratory for further analysis.

The basal stem and root tissues of the rootstocks were washed with tap water and rinsed with sterile distilled water. Margins of the lesions from the collar tissues were dissected longitudinally and in cross-sections, and the bark was removed before cutting into 0.5–1 cm^2^ pieces. These segments, including sapwood tissues, were surface-sterilized in 90% ethanol for 10 s, blotted dry on sterile filter papers, and transferred (five pieces per dish) to potato dextrose agar (PDA; HiMedia, Thane, India) and CMA-PARP medium [13] amended with 0.02 g L^−1^ benomyl (CMA-PARPB (Becton Dickinson, Sparks, MD, USA)). The Petri dishes were incubated at 24 °C in the dark for 72 h and visually checked for developing colonies over seven days. Hyphal tips of the resultant colonies were subcultured on fresh PDA to obtain pure single-hyphal cultures and determine colony morphology.

### 2.2. Morphological and Physiological Characterization

To determine the morphological characteristics of the isolates, they were plated onto potato dextrose agar (PDA) medium (HiMedia, Thane, India) and incubated at 25 °C for 5 days under mixed white and near-UV light with a 12 h photoperiod. Measurements of the fungal structures were performed by removing an agar square from the synthetic nutrient-poor agar (SNA) plates, placing it on a microscope slide, and adding a drop of water and a coverslip. Observations were performed using a Better Scientific Q190A-LCD (Better Scientific, Nanjing, China) microscope with differential interference contrast, and images were captured with the integrated 8 MP digital camera. Thirty measurements were obtained for each structure.

Cardinal growth temperatures were determined by inoculating PDA plates of 90 mm in diameter with a plug cut of 5 mm in diameter from the edge of an actively growing fungal colony. Two representative isolates were used in this study: three replicates of each isolate and temperature were prepared, and the experiment was performed twice. Colony growth was recorded after seven days in two orthogonal directions. Temperature growth experiments were performed at 5–40 °C in 5 °C intervals.

The temperature response of radial mycelial growth was described for each isolate with the Ratkowsky model [14], an asymmetric thermal performance model suited to the characteristic asymmetry of fungal thermal responses. The model was fitted to the individual growth measurements (not to treatment means) by nonlinear least squares using the rTPC and nls.multstart packages [15], with biologically constrained parameter bounds and multiple starting values. Cardinal temperatures (Tmin, Topt, Tmax) were derived from the fitted model, and their 95% confidence intervals were obtained by stratified bootstrap resampling (2000 iterations). All analyses and visualizations were performed in R v. 4.x [16] using the ggplot2 package [17].

### 2.3. DNA Extraction

Selected fungal isolates, DVP-3 and DVI-16, obtained from symptomatic plant tissues and maintained as monosporic cultures for six days at 25 °C in the dark for subsequent molecular analyses were used. Total DNA was extracted using the E.Z.N.A. Plant Miniprep Kit (Omega Bio-tek, Doraville, GA, USA) following the manufacturer’s instructions. Before extraction, mechanical disruption of fungal mycelium was performed by adding four tungsten carbide beads (3 mm in diameter) (Qiagen, Hilden, Germany) and homogenization in FastPrep-24 5G (MP Biomedicals, Santa Ana, CA, USA) at 5 m/s for 20 s in two consecutive cycles.

### 2.4. PCR Amplification and Sequencing

Two gene regions of recognized taxonomic importance were amplified by polymerase chain reaction (PCR): the internal transcribed spacer region (ITS) together with the 5.8S rRNA gene using primers ITS1F (5′-CTTGGTCATTTAGAGGAAGTAA-3′) [18] and ITS4 (5′-TCCTCCGCTTATTGATATGC-3′) [19], and the histone H3 gene (*his3*) using primers CYLH3F (5′-AGGTCCACTGGTGGCAAG-3′) and CYLH3R (5′-AGCTGGATGTCCTTGGACTG-3′) [20]. Amplification reactions were performed in a Peltier Thermal Cycler-200 (MJ Research, Hercules, CA, USA) under the following conditions: initial denaturation at 94 °C for 3 min, followed by 35 cycles of denaturation at 94 °C for 30 s, annealing at 55 °C for 30 s, elongation at 72 °C for 45 s, and a final extension at 72 °C for 10 min. Amplification of both genes was verified by electrophoresis of the PCR products on a 1.5% agarose gel before sequencing (Figure S1).The resulting PCR products were purified and bidirectionally sequenced by Macrogen Inc., Sequencing Center (The Netherlands, Europe), using the same primer pairs previously used, thereby ensuring the production of high-quality, intact sequences for subsequent phylogenetic analyses.

### 2.5. Phylogenetic Analyses

Consensus sequences were assembled using Sequencher v. 5.3 software (Gene Codes Corporation, Ann Arbor, MI, USA) and subsequently subjected to homology searches in the NCBI GenBank database using the Basic Local Alignment Search Tool (NCBI BLAST web server) to perform preliminary taxonomic identification (Table S1). A comprehensive phylogenetic dataset of 59 sequences was compiled, including 19 reference sequences from the genus *Pleiocarpon* representing the three described species: *P. algeriense* (nine sequences), *P. strelitziae* (nine sequences), and *P. livistonae* (one sequence). Thirty-seven additional reference sequences from phylogenetically related genera within the Nectriaceae were included: *Neonectria* (10 sequences), *Ilyonectria* (12 sequences), *Dactylonectria* (9 sequences), *Cylindrocladiella* (2 sequences), *Campylocarpon* (1 sequence), *Thelonectria* (1 sequence), and *Thyronectria* (1 sequence). *Xenogliocladiopsis cypellocarpa* (CBS 133814) was used as the outgroup to root the phylogenetic tree. These reference sequences were selected based on recent phylogenetic studies of the Nectriaceae family [21,22], prioritizing sequences from type strains and representatives of proven taxonomic quality.

### 2.6. Sequence Alignment, Concatenated Analysis, and Phylogenetic Inference

Sequences were aligned in Geneious Prime v.2026.1 [23] using the MAFFT v.7 algorithm [24], and the resulting alignments were trimmed with GBlocks v.0.91b [25] to remove ambiguously aligned regions at the 5′ and 3′ ends and prevent biases in phylogenetic inference. To maximize phylogenetic resolution and statistical power, a concatenated analysis was performed by combining the two molecular markers in Geneious Prime [23], yielding a final matrix of 59 sequences totaling 803 aligned characters (ITS: 396; *his3*: 407). Phylogenetic reconstruction was performed using Bayesian inference with the MrBayes plugin in Geneious Prime [23,26]. The concatenated ITS–*his3* dataset was partitioned by locus, and the GTR+I+Γ nucleotide substitution model was applied independently to each partition. Model parameters were unlinked across partitions, and relative rates among partitions were allowed to vary. Markov chain Monte Carlo (MCMC) analyses were run for 5,000,000 generations using four chains, with a heating temperature of 0.1 and sampling of all 1000 generations. The first 25% of sampled trees were discarded as burn-in, and convergence was assessed by verifying that the average standard deviation of split frequencies was below 0.01. The consensus tree was rooted with *Xenogliocladiopsis cypellocarpa*, and Bayesian posterior probabilities ≥ 0.70 were reported as nodal support values.

### 2.7. Pathogenicity Tests

Pathogenicity tests were conducted in a greenhouse from December 2024 to April 2025. One-year-old dormant vines cv. Red Globe grafted onto Salt Creek (Ramsey, *Vitis champini*) rootstock were used as plant material. These had previously been exposed to thermotherapy (50 °C for 30 min). The treated plants grew in plastic pots (30 cm in diameter × 40 cm in depth) filled with a sterile potting mix (75% peat, 25% sand, vol/vol). Inoculations began when the plants developed an active 15 cm shoot.

Two representative isolates of *Pleiocarpon* served as inoculum: DVP-3 (from grapevine collar rots in the Piura region) and DVI-16 (from grapevine collar rots in the Ica region). These isolates were selected according to the two principal grape-producing regions of Peru (Piura and Ica), and also because they exhibited representative colony morphology, and showed vigorous growth in culture.

The experiment comprised two runs: the first from December 2024 to February 2025, and the second from February to April 2025. Plants were inoculated by wounding. The procedure followed methods used for *Pleiocarpon* pathogenicity tests in grapevine [21], avocado [22], and other *Cylindrocarpon*-like asexual morphs on grapevine [27]. For each isolate, 20 plants were stem-inoculated by 8 cm from the rootstock base. A bark disk with a diameter of 5 mm was removed using a cork borer to expose the cambium. A PDA disk of similar size, colonized by *Pleiocarpon* mycelium, was placed on the exposed cambium. After inoculation, the wound was moistened with sterile water and sealed with a parafilm strip. The inoculated wounds of 20 plants per treatment were buried 8 cm deep in the substrate. Twenty control grapevine plants received uninfested PDA disks and were arranged as described above. The inoculated plants were kept on benches in a greenhouse and watered as needed. Greenhouse temperatures during the trial ranged from 26 °C to 32 °C. Symptoms of shoot wilt and other foliar changes were observed weekly.

After 6 weeks, plants from all treatments were removed from the pots to record lesions. The stem bark surface was scraped with a sterile scalpel. This revealed the margins between healthy and necrotic tissues, both above and below the inoculation point. The canker area was traced onto a transparent plastic sheet and then digitized. Quantification was done using Assess software 2.2 (American Phytopathological Society, St. Paul, MN, USA). The lesion area was calculated by subtracting the size of the inoculation wound. These data were used for statistical analyses.

To confirm that each inoculated *Pleiocarpon* isolate caused the lesions, small wood pieces were taken from the leading edges of the lesions. These were plated onto PDA medium to re-isolate the pathogen and confirm Koch’s postulates.

### 2.8. Data Analysis

Analysis of variance was performed using a completely randomized design, with lesion area recorded for each *Pleiocarpon* isolate in the pathogenicity tests. Means were separated according to Fisher’s least significant difference test (*p* < 0.05) using SAS statistical software version 9.0 (SAS Institute, Cary, NC, USA).

## 3. Results

### 3.1. Symptoms Description, Field Survey and Isolations

**Figure 1:**
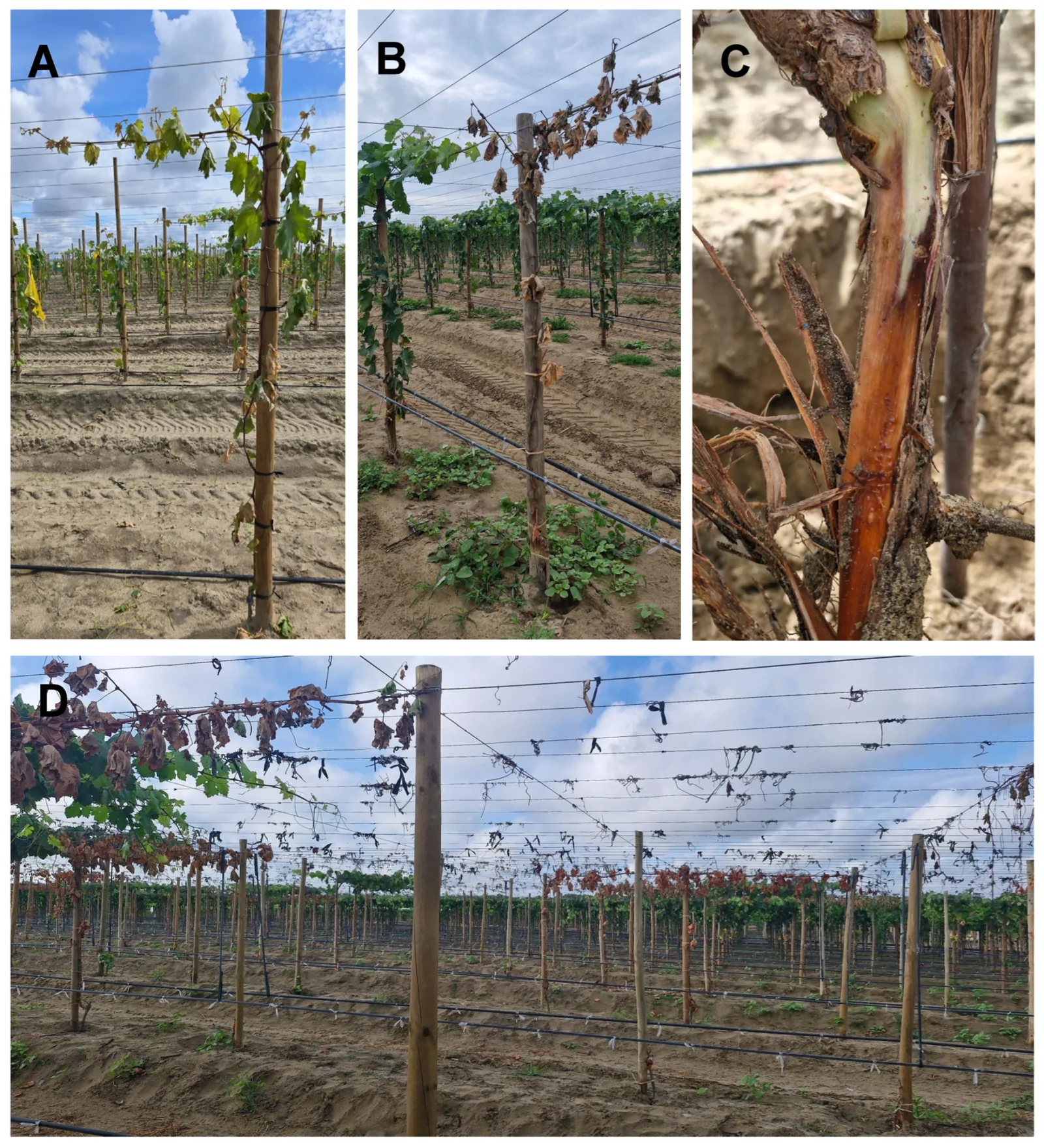
Field symptoms, collar rot necrosis, and extensive plant mortality on young grapevines. (**A**) Early decline is characterized by leaf wilting and shoot dieback in a newly established vineyard. (**B**) Advanced decline with severe dieback and canopy collapse. (**C**) Belowground collar rots along the rootstock after removal of the outer bark. Dark brown discoloration of cortical and vascular tissues is visible at the crown and proximal roots, consistent with infection by *Pleiocarpon* sp. (**D**) Extensive plant mortality a few days after the onset of decline symptoms.

Symptomatic plants were found on 1–2-year-old plantations. The first symptoms appeared as necrotic leaf margins on the oldest leaves. Affected vines soon showed sudden shoot wilting (Figure 1) and later, death of the aerial parts (Figure 1A,B).

Diseased plants showed no internal lesions on the shoots, cordons, or stems above the graft union. To better understand these symptoms, the soil around the stem was removed to expose the rootstock collar and lateral roots.

After removing the superficial tissues here, belowground collar rots of varying extents became visible along the rootstock. Lesions began in the buried portion, spreading toward the graft union and to the roots (Figure 1C). Within a few weeks, extensive areas of plants died after the initial symptoms (Figure 1D), requiring replanting. However, these new plants also died from the same disease in subsequent warm seasons.

Notably, approximately 95% of the symptomatic plants were from recent plantings less than 1 year old. Furthermore, all symptomatic plants (100%) had Salt Creek as their rootstock. A total of 44 fungal strains with similar characteristics were isolated from the infected plants.

In 2022, the first known outbreak of this disease affected over 10% of the plants. In 2023, six young commercial plantations experienced this syndrome. Disease incidence increased over time. In some vineyards, plant mortality reached up to 30%. Additionally, this disease first appeared, to a lesser extent, in the Ica region (southern Peru) in 2023. In 2024 and 2025, several other young commercial grapevine plantations were established. Symptomatic plants affected by this syndrome are commonly observed during late spring, summer, and early autumn (October–April).

### 3.2. Morphological and Physiological Characterization

Isolations from necrotic lesions of the collar rootstock consistently yielded a *Cylindrocarpon*-like anamorph, a group of fungi known to cause diseases such as black foot disease in grapevines and other woody plants. Under the isolation conditions used here, no *Phytophthora* spp. or other previously reported grapevine pathogens were recovered, although a semiselective medium for oomycetes was included in the isolation scheme. Recovered isolates had the following characteristics.

*Culture characteristics:* The mycelium was cottony, with average density on PDA. The surface was cinnamon to honey in color, with buff aerial mycelium. Zonation was absent, with homogeneous transparency and margin uneven on PDA. Reverse on PDA was similar to the surface. To expand upon these initial observations, the following mycological characteristics were documented for further comparative analysis.

**Figure 2:**
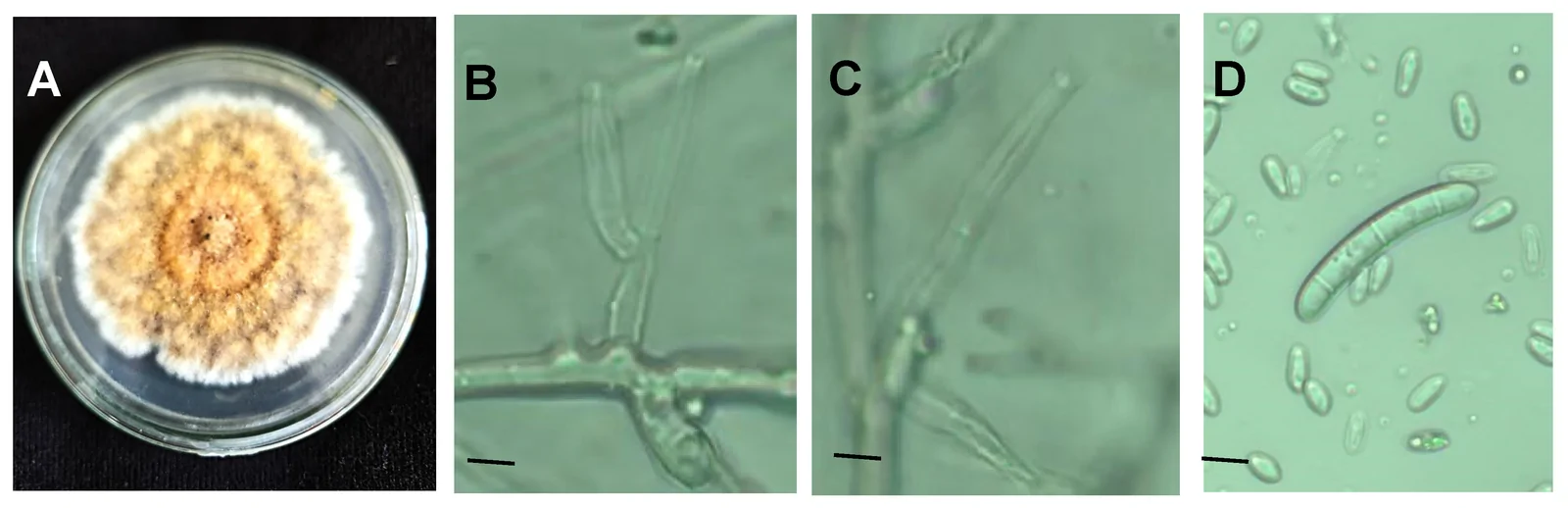
*Pleiocarpon* sp lineage. (**A**) Pattern of colony. (**B**,**C**) Simple conidiophores on aerial mycelium. (**D**) Macroconidia and microconidia. Bars = 10 μm.

*Description*: Ascomata were not observed. Conidiophores were simple or aggregated to form sporodochia. Simple solitary conidiophores, arising laterally or terminally from aerial mycelium to loosely aggregated, unbranched or sparsely branched 1–3-septate, were 46–95 μm long and bore one to two conidiogenous cells. Conidiogenous cells were monophialidic and cylindrical, tapering slightly towards the apex, with a length of 16–40 μm, a width of 2–3 μm at the base, and 1.5–2 μm near the apex (Figure 2). Measurements of microconidia aseptate, which is ellipsoid to ovoid or subcylindrical and straight to slightly curved, were (6−)7–8.5 (−9,3) × (2−)3–3.4 (−4) μm (av. 8.1 × 3.3 μm). Macroconidia hyaline were straight to curved; 1–5-septate was predominantly 3-septate, with the apex or apical cell typically slightly bent to one side and minutely beaked, and a base with sometimes visible, centrally located or laterally displaced hilum; measurments for 3-septate were (32−)43.3–48(−59) × (5−)6.5–7.3(−8.2) μm (av. 45.1 × 7.6 μm). Chlamydospores were absent.

**Figure 3:**
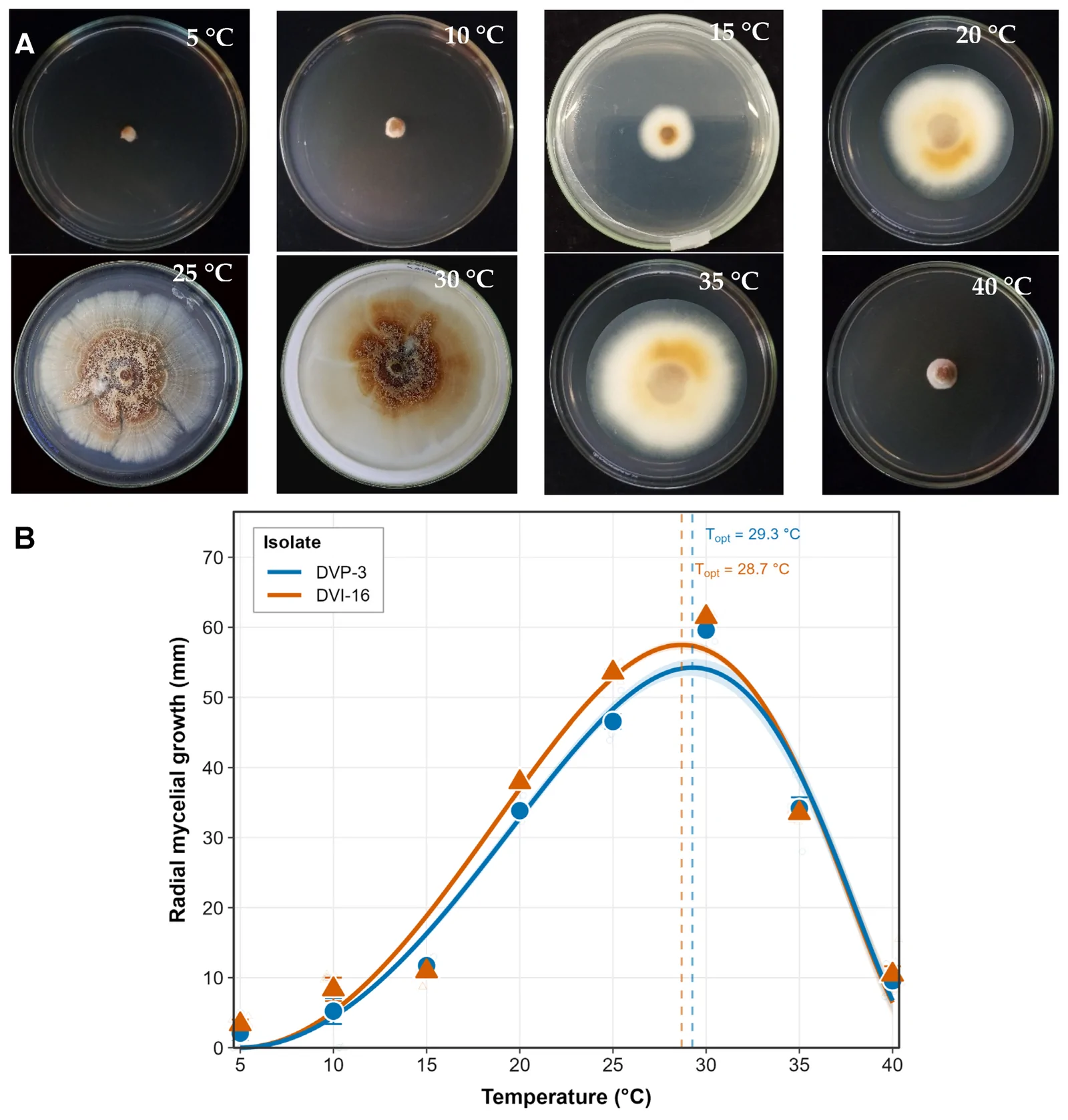
Influence of temperature on the radial mycelial growth of *Pleiocarpon* isolates DVP-3 and DVI-16. (**A**) Representative colony morphology grown on potato dextrose agar (PDA) after 7 days of incubation at temperatures ranging from 5 to 40 °C. (**B**) Effect of temperature on the mean radial mycelial growth (mm). Data points represent mean values for DVP-3 (blue circles) and DVI-16 (orange triangles). Solid lines represent the fitted Ratkowsky thermal performance models, and shaded areas indicate the 95% bootstrap confidence bands. Dashed vertical lines indicate the calculated optimum temperatures ($T_{opt}$): DVP-3 = 29.3 °C (95% CI: 28.9–29.5 °C); DVI-16 = 28.7 °C (95% CI: 28.6–28.8 °C).

*Cardinal growth temperatures:* The results showed that mycelial growth of *Pleiocarpon* isolates DVP-3 and DVI-16 exhibited a unimodal, asymmetric response to temperature. No growth was observed at 5 °C for DVP-3, whereas DVI-16 showed minimal development at this temperature. Growth increased progressively from 10 to 30 °C in both isolates, reaching a maximum at 30 °C, and then declined sharply at 35 and 40 °C (Figure 3).

The Ratkowsky model described the temperature response of both isolates closely (pseudo-R^2^ = 0.956, RMSE = 4.21 mm for DVP-3; pseudo-R^2^ = 0.947, RMSE = 4.84 mm for DVI-16). For isolate DVP-3, the fitted equation was √Y = 0.4380 (T − 4.95) [1 − exp(0.0923 (T − 42.00))], and the estimated optimal temperature (Topt) was 29.3 °C (95% bootstrap CI: 28.9–29.5 °C). For isolate DVI-16, the fitted equation was √Y = 0.4888 (T − 4.95) [1 − exp(0.0794 (T − 42.03))], with Topt = 28.7 °C (95% bootstrap CI: 28.6–28.8 °C).

Both isolates exhibited similar thermal optima near 29 °C, consistent with the maximum colony growth observed at 30 °C within the evaluated range. Because measurable growth persisted at 40 °C and ceased above it, the upper cardinal temperature lies just beyond the highest temperature tested and could not be estimated within the experimental range. Any value reported for Tmax should therefore be regarded as an extrapolation. Morphological and physiological features of the isolates matched with previously described *Pleiocarpon* species.

### 3.3. Molecular Identification and Phylogenetic Analysis

Single amplicons of the expected size were obtained for the ITS region and the *his3* gene in both isolates (Figure S1). Preliminary BLASTN searches placed DVP-3 and DVI-16 within *Pleiocarpon*, with the closest ITS match to *P. algeriense* CBS 144964 (99.29% identity) and the closest *his3* match to *P. strelitziae* CPC 27628 (98.77% for DVP-3 and 98.76% for DVI-16) (Table S1).

**Figure 4:**
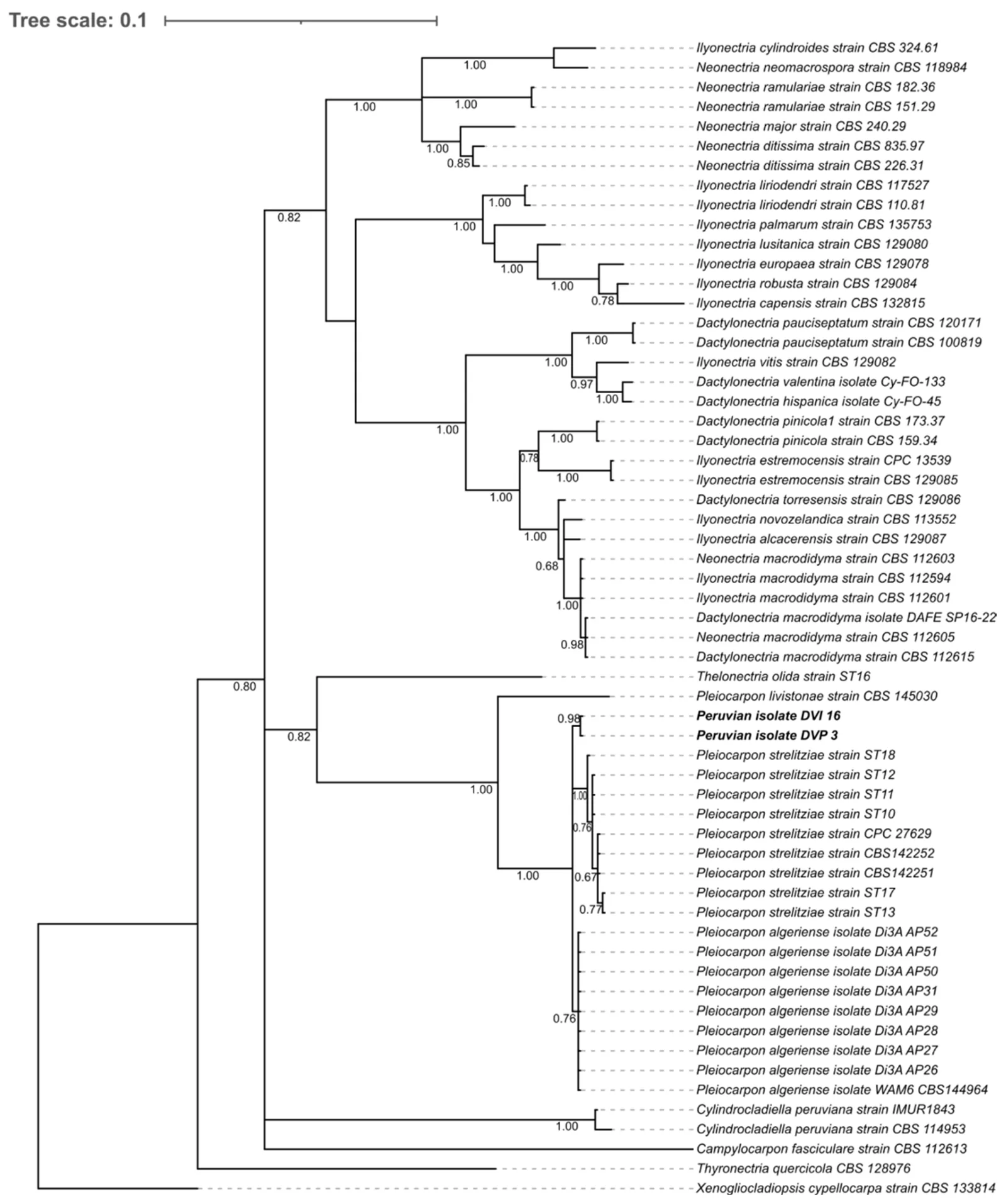
Bayesian phylogram inferred from the concatenated alignment of the nuclear ribosomal internal transcribed spacer (*ITS*) region and the histone H3 (*his3*) gene for the Peruvian isolates DVI-16 and DVP-3 and representative taxa of *Pleiocarpon* and closely related genera. Bayesian posterior probabilities (BPP ≥ 0.70) are shown at the nodes, and the Peruvian isolates analyzed in this study are indicated in bold. Branch lengths are proportional to the expected number of substitutions per site; the scale bar represents 0.1 expected substitutions per site. The tree was rooted using *Xenogliocladiopsis cypellocarpa* CBS 133814 as the outgroup.

Bayesian phylogenetic analysis of the concatenated *ITS*–*his3* matrix recovered the recognized *Pleiocarpon* species as well-supported, mutually exclusive clades. The Peruvian isolates DVP-3 and DVI-16 formed a strongly supported clade (PP = 0.98) that was resolved as sister to the *P. strelitziae* clade (PP = 1.00). This Peruvian and *P. strelitziae* group, together with *P. algeriense*, were placed within a strongly supported clade of *Pleiocarpon* (PP = 1.00), whereas *P. livistonae* was recovered as the next successive sister lineage to that clade (PP = 0.82). *P. algeriense* was resolved as a separate, well-supported clade (PP = 0.76) positioned more distantly within the genus (Figure 4).

The Peruvian isolates did not nest within any of the three described species, but formed an independent, well-supported branch. Short branches separated isolates within each species clade, whereas longer branches separated the major lineages, indicating low intraspecific and higher interspecific divergences across the dataset. Nucleotide sequences for DVP-3 and DVI-16 isolates, *ITS* (PZ158632, PZ158633) and *his3* (PZ234412, PZ234413), derived from this study were deposited in the NCBI GenBank database with their corresponding accession numbers to facilitate future comparative studies.

### 3.4. Pathogenicity Tests

All *Pleiocarpon* isolates tested were pathogenic to Salt Creek rootstock. In the first assay, DVP-3 caused shoot wilting in two of 20 plants at 35 days after inoculation (dai), increasing to 60% (12/20) by 42 dai, with declining symptoms. The DVI-16 isolate affected seven of 20 plants (35%) at 42 dai. In the second assay at 42 dai, shoot wilting was observed in 85% (17/20) of plants with DVP-3 and 55% (11/20) with DVI-16.

On the assessment date for both trials, irregular dark lesions were observed on the wood under the bark, starting from the point of inoculation on the rootstocks. *Pleiocarpon* isolates were consistently recovered from all inoculated plants, thereby confirming the Koch postulates, whereas no isolates were recovered from negative controls in either trial.

The inoculation assays revealed statistically significant differences among the treatments in both experimental assessments; however, no statistically significant differences were detected between the two assayed isolates. In the first inoculation assay, treatment effects were highly significant (*p* = 0.0001). In the second assay, the effects remained significant (*p* = 0.0032). This consistency showed a stable response pattern across the experiments.

At 42 dai, no lesions were observed in the control treatment (T0) in either assay, confirming the absence of disease under non-inoculated conditions. In contrast, treatments T1 (DVP-3) and T2 (DVI-16) developed measurable lesions. In the first assay, lesion areas reached 6.4 cm^2^ for T1 and 5.7 cm^2^ for T2; in the second, they increased slightly to 7.1 cm^2^ and 6.3 cm^2^, respectively.

**Figure 5:**
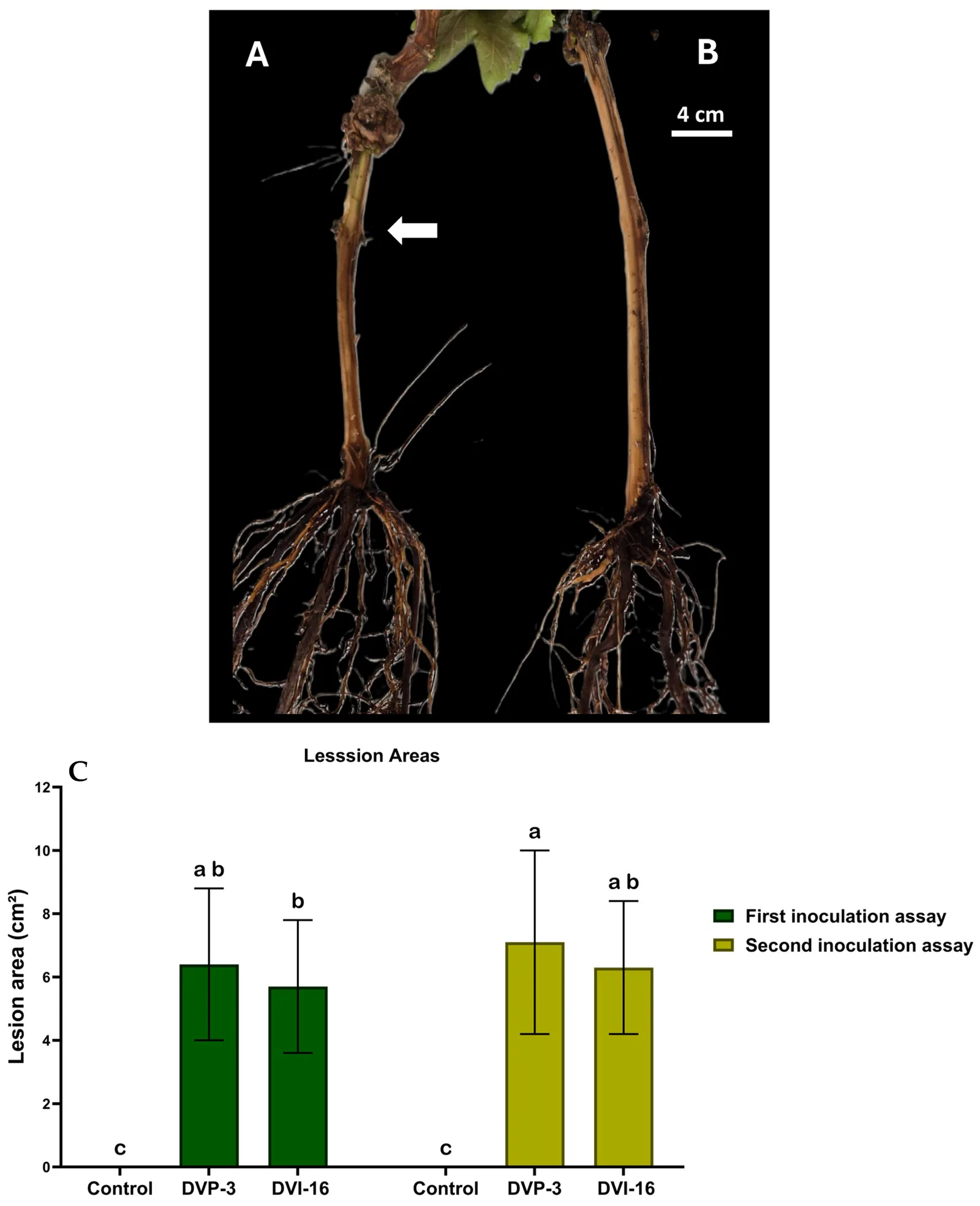
Pathogenicity of *Pleiocarpon* isolates on ‘Salt Creek’ grapevine rootstock. (**A**) Rootstock inoculated with a pathogenic *Pleiocarpon* isolate; arrow indicates lesion development. (**B**) Non-inoculated control rootstock; scale bar is 4 cm. (**C**) Lesion area of *Pleiocarpon* isolates DVP-3 and DVI-16 on grapevine rootstock Salt Creek (Ramsey—*Vitis champini*) under greenhouse conditions. Bars represent the mean lesion area (cm^2^) from two independent assays (*n* = 20 per treatment). Error bars indicate the standard deviation. Means followed by the same letter are not significantly different according to Fisher’s protected LSD test (*p* < 0.05). Control plants (T0) were inoculated with non-infested PDA disks and did not develop any symptoms.

According to the LSD test, both inoculated treatments differed significantly from the control; however, they did not differ significantly from each other, as indicated by the shared significance letters (Figure 5). This indicates that DVP-3 and DVI-16 exhibited similar levels of aggressiveness or similar pathogenic responses under the evaluated conditions.

The average lesion area increased from 4.0 cm^2^ in the first assay to 4.4 cm^2^ in the second assay, reflecting consistent disease expression across experiments. Furthermore, the coefficients of variation were low to moderate (7.67% and 12.60%), indicating acceptable experimental exactness and measurement reliability.

## 4. Discussion

### 4.1. Etiology of an Emerging Collar-Rot Syndrome in Young Grapevines

This study documents a severe and recurrent decline of young grapevines in commercial vineyards in Peru. The disease is associated with belowground collar rot caused by an aggressive *Pleiocarpon* sp. The syndrome represents an atypically severe form of black foot disease, characterized by rapid plant mortality soon after vineyard establishment, and is most evident in plantings made during the warmest months (November–March), with major economic consequences that include plant loss, replanting, and reduced stand uniformity.

Field observations, morphological analysis, cardinal-temperature testing, phylogenetic inference, and pathogenicity assays converge in implicating a *Pleiocarpon* sp. as the causal agent of this emerging decline syndrome. A comparable emergence of black foot pathogens in young vineyards was recently documented for *Campylocarpon fasciculare* in China, where greenhouse inoculation likewise reproduced wilting and vine death [28], underscoring that newly recognized Nectriaceae can act as primary pathogens of recently established vines.

Typically, black foot disease is associated with soilborne fungi in the Nectriaceae, including *Campylocarpon*, *Cylindrocladiella*, *Cylindrodendrum*, *Dactylonectria*, *Ilyonectria*, *Neonectria*, *Thelonectria*, and *Pleiocarpon* [21,29]. These fungi generally infect roots and basal tissues, causing necrosis, reduced root biomass, stunted growth, chlorosis, and eventually vine death [30,31,32]. However, the main evidence of our study is that the collar rots observed were more extensive and rapidly lethal than previously reported, denoting a more aggressive disease phenotype associated with this pathogen.

The significance of *Pleiocarpon* as a pathogen is emphasized by its impact on diverse cropping systems. For example, *P. strelitziae* was first described, causing basal stem and root rot of *Strelitzia reginae* in Italy [33], and *P. algeriense*, initially isolated and described from grapevine nurseries in Algeria [21], was later reported as the causal agent of stem and crown rot of avocado (*Persea americana*) under field conditions [22]. These reports emphasize the genus’s capacity to induce extensive necrosis, structural collapse of crown tissues, and rapid plant death. In the context of our research, the present study expands the documented host–pathogen interaction of *Pleiocarpon* to commercial grapevines in South America and supports the presence of a distinct lineage associated with severe vineyard decline.

The Peruvian isolates exhibited morphological features that are largely consistent with those previously described for *P. algeriense* and *P. strelitziae*, including colony pattern, conidiophore organization, conidial morphology, and the absence of chlamydospores. These overlapping phenotypic traits are not unexpected, as members of *Pleiocarpon* belong to the *Cylindrocarpon*-like fungi, a group characterized by high morphological conservatism and limited diagnostic value of traditional phenotypic characters. Indeed, the original descriptions of *P. strelitziae* and *P. algeriense* relied on a combination of morphology and multilocus phylogenetic analyses because morphological features alone were insufficient to discriminate species within the genus [21,33]. Consequently, although the Peruvian isolates are morphologically comparable to the previously described *Pleiocarpon* species, their taxonomic placement is supported primarily by molecular phylogenetic evidence rather than by unique morphological characters.

### 4.2. Phylogenetic Placement of the Peruvian Pleiocarpon Isolates

To further characterize the pathogen, phylogenetic analyses based on concatenated ITS and *his3* loci consistently resolved the Peruvian isolates within *Pleiocarpon*, with the isolates DVP-3 and DVI-16 forming a well-supported sister lineage to *P. strelitziae*. The concatenated two-locus analysis recovered the Peruvian isolates as a well-supported lineage within the genus, consistent with the phylogenetic backbone recently established for the Nectriaceae [34]. Comparable geographically structured lineages have been reported among other black foot–associated fungi, where they sometimes correspond to cryptic species diversity [21,35]. Because the present placement rests on only two loci (ITS and *his3*) and the isolates show high sequence identity to described species, the molecular evidence is interpreted here as consistent with a distinct lineage within *Pleiocarpon* rather than as the basis for a formal species. A robust species delimitation would require additional loci (e.g., *tef1* and *tub2*) and a dedicated taxonomic treatment, which lie beyond the scope of this study; the pathogen is therefore referred to throughout as *Pleiocarpon* sp.

These phylogenetic results are informed by the molecular genes chosen. Within *Cylindrocarpon*-like fungi, ITS sequences alone frequently provide insufficient resolution to delimit species boundaries, mainly within genera characterized by high morphological conservatism and cryptic diversity [35,36,37]. The histone H3 gene (*his3*) has proven more discriminating for this group: the genus *Pleiocarpon* was erected by Aiello et al. [33] for *P. strelitziae* based on a multilocus phylogeny that incorporated *his3*, and in the subsequent description of *P. algeriense* from Algerian grapevine nurseries, Aigoun-Mouhous et al. [21] reported that LSU and *rpb2* did not separate *P. algeriense* from *P. strelitziae*, whereas *his3*, together with ITS, *tef1* and *tub2*, provided diagnostic differences between the two species.

Moreover, the usefulness of this marker extends to routine species identification: Akgül et al. [38] characterized seven black foot species across four genera in Turkish nurseries using *his3* as the sole molecular marker, employing the same *CYLH3F/CYLH3R* primer pair used in the present study. This demonstrates that this locus provides species-level discrimination sufficient for applied diagnostic and epidemiological purposes. Accordingly, in the present work, the combination of ITS and *his3* into a concatenated dataset strengthened phylogenetic inference beyond what either marker could achieve independently, and produced a robust placement of the Peruvian isolates within *Pleiocarpon*.

The concatenated ITS–*his3* phylogeny consistently resolved the Peruvian isolates as a well-supported lineage within *Pleiocarpon*, sister to *P. strelitziae*, but this level of evidence should be interpreted in light of the objectives of the present study. Our primary aim was to identify the causal agent responsible for an emerging collar rot disease of young grapevines rather than to undertake a formal taxonomic revision of the genus. For this purpose, the combination of ITS and *his3* provided sufficient phylogenetic resolution to demonstrate that the pathogen belongs to *Pleiocarpon* and represents a genetically distinct lineage associated with the disease. Nevertheless, robust species delimitation within Nectriaceae generally requires multilocus datasets incorporating additional informative loci such as *tef1*, *tub2*, *rpb2*, and LSU, together with broader geographic sampling and detailed comparative analyses. Such an integrative taxonomic approach falls beyond the scope of the present work but represents an important direction for future studies aimed at resolving the evolutionary relationships and formal taxonomic status of the Peruvian lineage.

### 4.3. Thermal Response of the Pathogen and Vineyard Establishment

In Peru, commercial table grape production relies almost exclusively on Salt Creek (Ramsey, *Vitis champini*) rootstock, valued for its tolerance to salinity, sandy soils, drought, and nematodes, and for its compatibility with commercial scions [39,40,41]. This near-exclusive use is relevant here because all documented cases occurred on this single rootstock, which may amplify disease incidence and regional spread. Our data, however, do not allow Salt Creek to be ranked as more or less susceptible than other rootstocks, since no comparative trial was performed, and relative susceptibility to black foot pathogens depends strongly on inoculum pressure [42].

All tested *Pleiocarpon* isolates were pathogenic on Salt Creek, confirming that this rootstock is a competent host. Because black foot pathogens infect basal tissues mainly through wounds caused by grafting, transplanting, or early root growth [30], the wound-inoculation method was appropriate to confirm pathogenicity, although it was not intended to reproduce field epidemiology; this approach follows established pathogenicity protocols for *Pleiocarpon* and other *Cylindrocarpon*-like fungi on grapevine [21,27]. The similar aggressiveness of isolates from the two producing regions further suggests that host and environmental factors are more influential than isolate-specific variation.

The thermal response of the pathogen links these observations to the conditions under which the disease is expressed. New vineyards are established between October and February, when warm soils promote rapid vine growth but also coincide with the period of greatest disease expression. Consistent with this, the estimated optima of the Peruvian isolates (T_opt_ 28.7 to 29.3 °C; Ratkowsky model) and the maximum growth near 30 °C indicate adaptation to the elevated soil temperatures typical of coastal Peru during establishment. Rapid mycelial growth at these temperatures may enhance colonization and infection success [43], which, together with the repeated occurrence of collar infections in buried basal tissues, may help explain the rapid progression and high mortality observed.

### 4.4. Implications for Disease Diagnosis, Nursery Sanitation, and Vineyard Management

The epidemiology of this pathogen remains poorly understood, due in part to the absence of recognized long-term survival structures such as chlamydospores. Infected nursery material is therefore a plausible primary inoculum source, which highlights the importance of transplant-associated wounds; consistent with this, black foot pathogens have been recovered from symptomless grapevine nursery stock, where they can remain latent until planting [44]. Spread across grape-growing regions can be explained by the exchange of plant material among production areas, with infected nursery stock being a well-documented pathway for the dissemination of black foot pathogens [45].

An interesting aspect emerging from the phylogenetic analyses is the close relationship between the Peruvian lineage and *P. strelitziae*. The latter species was originally described from basal stem and root rot of *Strelitzia reginae* in Italy [33], a country where grapevine cultivation is widespread and extensively studied. Despite the long-standing importance of viticulture in Italy and the intensive investigation of grapevine trunk diseases, *P. strelitziae* has not been reported to have infected grapevine under natural conditions. This apparent absence may reflect host (rootstock) specificity, ecological separation, or simply limited sampling of this relatively uncommon species. Nevertheless, given its close phylogenetic affinity with the Peruvian grapevine isolates and its demonstrated pathogenicity on woody hosts, the possibility that *P. strelitziae* possesses pathogenic potential on grapevine cannot be excluded. Comparative pathogenicity assays involving multiple *Pleiocarpon* species and grapevine hosts would therefore be valuable for clarifying host range evolution and assessing the epidemiological significance of this lineage within grapevine production systems.

The origin of this *Pleiocarpon* sp. in Peru remains unknown and warrants further investigation. Its aggressiveness and its direct link to severe vineyard-establishment failures and recurrent infections are concerning, particularly where a single rootstock is grown under warm, arid conditions in the country. These observations point to a need for optimized diagnostic protocols, as early detection and accurate identification are essential to limit dissemination through planting material and to mitigate vineyard losses. Integrated disease management should focus accordingly on pathogen-free planting material, nursery hygiene, diversified rootstocks, and stress-reducing cultural practices. Additionally, establishing thermal thresholds could improve risk assessment and disease management in climate-conducive scenarios.

### 4.5. Study Limitations and Future Research Needs

Several boundaries delimit the scope of these findings. Phylogenetic placement and pathogenicity were established using two representative isolates (DVP-3 and DVI-16) out of the larger collection recovered in the field; while these isolates were consistent in morphology, thermal response, and aggressiveness, a broader sampling would better capture intraspecific variation. The two-locus dataset (ITS and *his3*) is adequate to position the Peruvian isolates within *Pleiocarpon* and to recognize them as a distinct lineage, but it is not sufficient for a formal species description, which would require additional loci and a dedicated taxonomic treatment. Because the inoculation trials did not include alternative rootstocks, the susceptibility of Salt Creek relative to other genotypes remains undetermined. Finally, the disease-occurrence figures reported here were derived from field observations rather than from a longitudinal epidemiological survey designed for incidence estimation, and should be read as indicative of disease pressure. Future work should combine multilocus or genomic analyses to resolve the taxonomic status of the lineage, comparative rootstock assays to assess relative susceptibility, and structured monitoring to quantify incidence and characterize the conditions that govern infection and symptom onset.

## 5. Conclusions

A *Pleiocarpon* sp. is identified as the causal agent of an emerging collar rot that drives rapid decline and death of young table grapevines grafted onto Salt Creek rootstock in coastal Peru. Greenhouse inoculations reproduced collar lesions and shoot wilting and fulfilled Koch’s postulates, and the warm thermal optimum of the isolates is consistent with the conditions that prevail during vineyard establishment in the region. The Peruvian isolates form a distinct lineage within *Pleiocarpon*, close to *P. strelitziae*, although their formal taxonomic status remains to be resolved. From a horticultural standpoint, the recurrence of the disease and the near-exclusive reliance on a single rootstock highlight the need for early diagnosis, the use of pathogen-free nursery material, nursery sanitation, and preventive management during the establishment of young vineyards under warm coastal conditions.

## Data Availability

The datasets generated and analyzed during the current study are available from the corresponding authors upon reasonable request. Raw sequencing data supporting the molecular identification results have been deposited in GenBank.

## Supplementary Materials

The following supporting information can be downloaded at https://www.mdpi.com/article/10.3390/ijms27167448/s1.

## Abbreviations

The following abbreviations are used in this manuscript:

DAI	Days after inoculation
ITS	Internal transcribed spacer
*his3*	Histone H3
PDA	Potato dextrose agar
PCR	Polymerase chain reaction
ANOVA	Analysis of variance
SD	Standard deviation
DNA	Deoxyribonucleic acid
