# Supplementary material for: Emerging Collar Rot of Young Table Grapevines Associated with Pleiocarpon sp. in Coastal Peru
Source: Int J Mol Sci. 2026 Aug 20;27(16):7448. doi: 10.3390/ijms27167448 (PMC13513269; doi:10.3390/ijms27167448)
Supplement: Supplementary file 1 [file ijms-27-07448-s001.zip › ijms-4408126-supplementary.pdf]

SUPPLEMENTARY MATERIAL

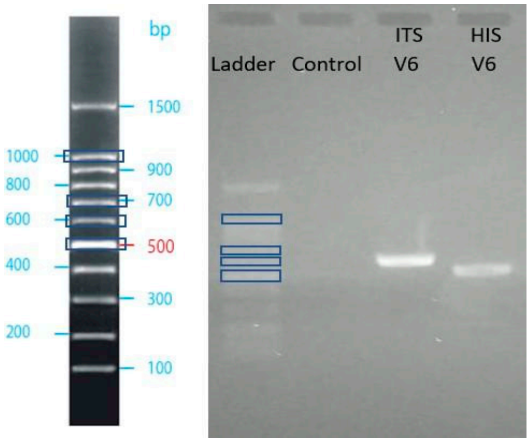

Fig. S1. Agarose gel electrophoresis (1.5%) showing the PCR amplification products of the internal transcribed spacer (ITS) region and the histone H3 (*his3*) gene for *Pleiocarpon* isolate DVP-3. Lane 1: 100 bp DNA ladder (ranging from 100 to 1,500 bp; the 500 bp reference band is indicated); Lane 2: Negative control (nuclease-free water); Lane 3: ITS region amplified with primers ITS1F/ITS4 (approx. 450–500 bp); Lane 4: *his3* gene amplified with primers CYLH3F/CYLH3R (approx. 380–400 bp).

Table S1. Top NCBI BLASTN hit for *Pleiocarpon* isolate DVP-3 and DVI-16 based on the internal transcribed spacer (ITS) region and the histone H3 (*his3*) gene.

Isolate	Gene region	Top BLAST match	Accession no.	Query coverage (%)	Identity (%)
DVP-3	ITS	*Pleiocarpon algeriense* CBS 144964 ITS region, type material	NR_164274.1	99	99.29
DVI-16	ITS	*Pleiocarpon algeriense* CBS 144964 ITS region, type material	NR_164274.1	99	99.29
DVP-3	*his3*	*Pleiocarpon strelitziae* strain CPC27628 histone H3 (*his3*) gene, partial cds	KY304616.1	100	98.77
DVI-16	*his3*	*Pleiocarpon strelitziae* strain CPC27628 histone H3 (*his3*) gene, partial cds	KY304616.1	99	98.76

Searches were performed against the NCBI core_nt database using BLASTN. Query lengths: ITS, 569 bp; *his3*, 487 bp.
